# Fluorescence Polarization Immunoassay for Rapid, Sensitive Detection of the Herbicide 2,4-Dichlorophenoxyacetic Acid in Juice and Water Samples

**DOI:** 10.3390/bios15010032

**Published:** 2025-01-09

**Authors:** Liliya I. Mukhametova, Marya K. Kolokolova, Ivan A. Shevchenko, Boris S. Tupertsev, Anatoly V. Zherdev, Chuanlai Xu, Sergei A. Eremin

**Affiliations:** 1 Faculty of Chemistry, M.V. Lomonosov Moscow State University, Leninsky Gory 1/3, 119991 Moscow, Russia; kolokolovamasha@yandex.ru (M.K.K.); ivansevcekno@gmail.com (I.A.S.); 2 A. N. Bach Institute of Biochemistry, Research Center of Biotechnology of the Russian Academy of Sciences; Leninsky Prospect 33, 119071 Moscow, Russia; zherdev@inbi.ras.ru; 3 N.N. Semenov Federal Research Center for Chemical Physics, Russian Academy of Sciences, Kosygina 4, 119991 Moscow, Russia; btoupersev@gmail.com; 4 School of Food Science and Technology, Jiangnan University, Wuxi 214122, China; xcl@jiangnan.edu.cn

**Keywords:** immunosensing, on-site detection, fluorophores, fluorescence polarization, toxic contaminants, pesticides, 2,4-Dichlorophenoxyacetic acid, food safety control

## Abstract

2,4-Dichlorophenoxyacetic acid (2,4-D) is one of the popular herbicides that is widely used in agriculture and can be found in food and water. A rapid and sensitive fluorescence polarization immunoassay (FPIA) was proposed for the detection of 2,4-D in juice and water. New tracers, 2,4-D-buthylenediamin fluoresceinthiocarbamyl (2,4-D-BDF) and 2,4-D-glycine aminofluorescein (2,4-D-GAF), were obtained and characterized. Monoclonal antibodies (MAb) obtained against 2,4-D were used as a recognition reagent. The kinetics of the interaction of MAb and tracers were studied, and the kinetic parameters of their binding were calculated. High specificity of binding of tracers and MAb was shown. In this work, an approach was elaborated on to reduce the detection limit of 2,4-D by the FPIA method by changing the volume of the studied sample. The optimized FPIA in a competitive format was characterized by the LODs of 2,4-D 8 and 0.4 ng/mL and the working ranges 30–3000 ng/mL and 3–300 ng/mL for juice and water, respectively. The entire test cycle (from sample receipt to evaluation of the analysis results) took only 20 min. The test for the recovery of 2,4-D in juice and water gave values from 95 to 120%, which demonstrated the reliability of the herbicide determination in real samples.

## 1. Introduction

Modern agricultural technologies are inextricably linked with the application of pesticides (chemical plant protection products). 2,4-Dichlorophenoxyacetic acid (2,4-D) remains one of the most commonly used herbicides worldwide due to its low cost, selectivity, efficacy, and broad spectrum of weed control [1]. 2,4-D controls broadleaf weeds and other vegetation in pastures, lawns, golf courses, forests, roads, and parks [2]. 2,4-D is readily soluble in water, which facilitates its rapid penetration through leaves/roots and makes it a more effective herbicide. Over 1500 commercial herbicide products contain 2,4- D as an active ingredient [3]. 2,4-D is a moderately persistent chemical. However, it readily breaks down in fields into 2,4-dichlorophenol, a hazardous metabolite that can linger in the soil for a very long period [4,5].

The agricultural application of 2,4-D and the following use of the obtained production should be integrated with an understanding of the existing evidence that 2,4-D poses a risk to both human health and the environment [6,7,8]. Although human data are preferred, maximum tolerated dose (MTD) is often based on animal studies because appropriate human studies are lacking. The MTD determined for mammals depends not only on the animal species but also on the gender. For example, for male and female rats, MTD is 150 and 75 kg(^−1^) day(^−1^), respectively. The minimum toxic amount of 2,4-D for humans is 3 to 4 g or 40 to 50 mg/kg, and it has been demonstrated that ingesting 6.5 g can result in fatal outcomes in adults [9]. The acute lethal serum levels of 2,4-D are typically between 447 and 826 mg/L. 2,4-D belongs to endocrine-disrupting chemicals; it interferes with the action of estrogen, androgen, and thyroid hormones [10]. Furthermore, 2,4-D may reduce fertility and increase the risk of birth defects [11]. Air contaminated with 2,4-D can cause skin irritation, itching, burning, shortness of breath, and burning in the upper lungs, and its ingestion leads to vomiting, diarrhea, headache, skeletal muscle damage, irritation, hypertension, etc. [12,13]. In 2015, the International Agency for Research on Cancer declared 2,4-D a possible human carcinogen [14]. To exert its toxicological effect, 2,4-D is transported to the target organ via the bloodstream. Due to its amphiphilic nature, it can be transported either by lipoproteins, human serum albumin (HSA), or distributed across red blood cell membranes. Human serum albumin (HSA) is a well-known transporter in human circulation due to its ability to bind a large number of exogenous and endogenous compounds [15]. HSA has been shown to be able to bind to 2,4-D [16,17], which facilitates its distribution throughout the body and the spread of its toxic effect. The widespread use of 2,4-D, its toxicity, and cases of fatal poisoning necessitate the analysis of this herbicide in various samples.

In addition to the possible harmful effects on people, there are risks of contamination of the soil and nearby water bodies [18,19]. According to the Environmental Protection Agency, 2,4-D has been found not only in ground and surface water but also in drinking water [7,8].

According to the World Health Organization document, the content of 2,4-D in drinking water should not exceed 30 ng/mL [20]. The maximum permitted levels of 2,4-D in milk are 10 ng/mL [21,22]; in mammal meat, 200 μg/kg; in fruits (pomes), 10 μg/kg; in stone fruits, 50 μg/kg; in berries and small fruits, 100 μg/kg [21]; and in cereals, up to 2000 μg/kg. The necessity of 2,4-D control has also been stated by many national regulations [23]. For example, the Canadian guidelines on 2,4-D fixed its maximum acceptable concentration at 0.1 mg/L in terms of kidney toxicity in rats [24]. The same maximum concentration is set in Australia and New Zealand [25].

The situation described above requires accessible tools for wide and reliable detection of 2,4-D in foodstuffs and the environment. The most commonly used method for this purpose is HPLC in combination with UV or mass spectrometric detection [19,26]. However, they require expensive equipment, long sample time preparation, and highly qualified personnel. Electrochemical [27,28], spectrophotometric [29], and ELISA [13,14,30,31] methods are also used to detect 2,4-D. However, these methods also have limitations; for example, the ELISA is a multi-stage, labor-intensive, and time-consuming method.

Thus, the variety of techniques used allows for the determination of 2,4-D with sufficient sensitivity and selectivity, but there is an urgent need to conduct express tests outside the laboratories. A prospective solution to this task is fluorescence polarization immunoassay (FPIA), a rapid single-stage analytical technique [32,33].

**Figure 1:**
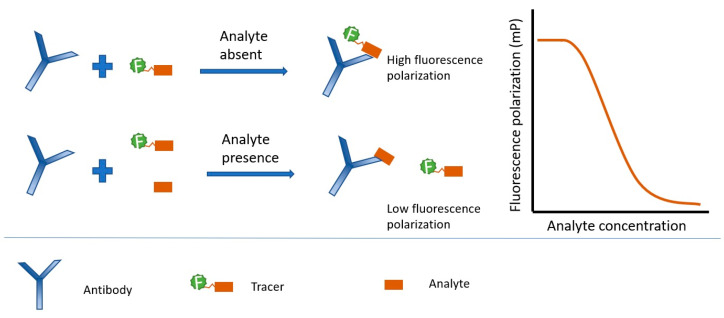
The principle of competitive FPIA.

FPIA is a homogeneous immunoassay that uses an antigen labeled with a fluorescent dye (tracer). Upon binding to the antibody, the free rotation of the tracer decreases, and the fluorescence polarization signal increases (Figure 1). The principle of competitive FPIA is based on the fact that in the presence of a free analyte, which competes with the tracer for binding sites on the antibody, the amount of tracer bound to the antibody decreases, and the FP signal decreases.

The existing applications of the FPIA technique for 2,4-D detection [34,35,36,37,38] demonstrated significant variability in detection limits, from 4 to 100 ng/mL, which is accomplished with various decisions concerning the used fluorescent derivative (tracer)—an antigen labeled with a fluorescent dye. The antigen and fluorescent label are connected by a linker, the length of which can also affect the specificity of the analysis [39]. Low-specificity recognition elements can lead to poor stability, low sensitivity, and high economic costs [40].

In this work, we obtained and characterized new conjugates of 2,4-D with glycine aminofluorescein (GAF) and with buthylenediamin fluoresceinthiocarbamyl (BDF) and studied their binding to a monoclonal antibody against 2,4-D (MAb). The conjugates had different spacer lengths between fluorescein and 2,4-D: one CH2 group for 2,4-D-GAF and four CH2 groups for 2,4-D-BDF. Changing the length of the spacer in the tracer can significantly affect the sensitivity of the assay [39]. Therefore, we decided to test how increasing the spacer would affect the sensitivity of the assay. The advantage of the GAF label is its commercial availability since this would promote the unification of preparations obtained in different laboratories. Previously described fluorescent dye ethylenediamine fluorescein was synthesized from the corresponding diamine and fluorescein isothiocyanate (FITC) [36].

As a homogeneous assay, FPIA is more susceptible to interference from matrix effects than other heterogeneous assays. Food samples may require different preparations depending on the complexity of the matrix. Sometimes, a simple dilution is sufficient, the multiplicity of which depends on the object being studied. In this work, studying the possibility of determining 2,4-D in a juice and water sample, an approach was taken to reduce the detection limit of FPIA by varying the volume of the sample being studied.

## 2. Materials and Methods

### 2.1. Reagents and Materials

2,4-dichlorophenoxyacetic acid (2,4-D), 2,4,5-chlorophenoxyacetic acid (2,4,5-T), 2,4-chloro-5-fluorophenoxyacetic acid (2,4,5-CFPA), 2,-chloro-4-fluorophenoxyacetic acid (2,4-CFPA), pentachlorophenoxyacetic acid (5-CPA), 4-chloro-o-toluoxyacetic acid (4-CTA), dimethylformamide, methanol, Na_2_B_4_O_7_, H_3_BO_3_, NaN_3_, N,N′-dicyclohexylcarbodiimide (DCC), N-hydroxysuccinimide (NHS), and buthylenediamine hydrochloride were acquired from Sigma-Aldrich Corporation (Saint Louis, MO, USA). The 5-(aminoacetamido)fluorescein (GAF) was obtained from Fisher Scientific (Wien, Austria). Silica Gel TLC ALUGRAM^®^Xtra SIL G/UV_254_ plates were produced by Merck (Macherey-Nagel, Düren, Germany). The syringe filters were produced by Agilent (Santa Clara, CA, USA). All other compounds were analytically pure. HPLC solvent water (H_2_O), acetonitrile (ACN), and formic acid (HCOOH) were obtained from Merck KGaA (Darmstadt, Germany).

Monoclonal antibodies against 2,4-D were obtained earlier at Jiangnan University, as described in [41]. The concentration of MAb was calculated by UV measurements based on the absorbance value obtained at 280 nm for 1 mg/mL solution, which is 1.3.

Quantification of analyte binding was assessed by measuring fluorescence polarization using a Sentry-200 portable reader (Ellie LLC, Germantown, WI, USA). Standard 10 × 75 mm borosilicate glass tubes were used in these assays. The instrument excitation wavelength λex was 485 nm, which was provided by a light-emitting diode (LED) source, and emission (λem) was detected at 535 nm using a photomultiplier tube. Data analysis was performed using SigmaPlot 11 (Systat Software Inc., Palo Alto, CA, USA).

### 2.2. Synthesis of Buthylenediamin Fluoresceinthiocarbamyl

The method outlined in [42] was applied to synthesize buthylenediamine fluoresceinthiocarbamyl (BDF). A total of 50 mL of methanol with 0.5 mL of triethylamine was used to dissolve 400 mg of buthylenediamine dihydrochloride (1.5 µmol). After 30 min of incubation, FITC (117 mg, 300 µmol) was added dropwise to the stirred reaction mixture after being dissolved in 10 mL of the same solvent. The final solution was stirred and incubated for one hour at room temperature in the dark before being stored for the entire night under the same conditions. After passing through a paper Watman filter, the orange precipitate that resulted from the synthesis was rinsed with 10 milliliters of methanol. The finished item was left to dry in the dark at room temperature.

### 2.3. Synthesis of 2,4-D–GAF and 2,4-D–BDF

The tracer synthesis was based on the protocol outlined in [36]. The molar ratio 2,4- D:DCC:NHS under the synthesis was 1:2:2. A total of 500 µL of DMFA was used to dissolve DCC (2.5 mg) and NHS (1.5 mg). After adding 2,4-D (5 mg), the mixture was incubated at room temperature for 18 h. After 5 min centrifugation of the reaction mixture at 10,000× *g*, the supernatant was gathered. The supernatant was then mixed with 15 mg of either BDF or GAF and 20 µL of trimethylamine, and it was left in the dark for 24 h. Following that, thin-layer chromatography (TLC) in a 4:1 chloroform–methanol ratio was used to separate compounds of the reaction mixture. Every yellow band was gathered and dissolved in methanol.

### 2.4. HPLC-MS Conditions

High-performance liquid chromatography coupled with mass spectrometry (HPLC-MS) analysis was performed using a Thermo Scientific Dionex UltiMate 3000 HPLC system interfaced with a Thermo Scientific Q Exactive Orbitrap mass spectrometer (Bremen, Germany). Separation was achieved using a Hypersil Gold aQ C18 reversed-phase column (2.1 × 100 mm, 3 µm particle size). The mobile phase consisted of a gradient elution employing two solvents: mobile phase A (0.1% formic acid in 5% acetonitrile/95% water) and mobile phase B (0.1% formic acid in 100% acetonitrile). The gradient profile was as follows: 0–1 min, 0% B; 1–15 min, 0–95% B; 15–18 min, 95% B; and 18–20 min, 0% B at a constant flow rate of 0.60 mL/min. The column temperature was maintained at 45 °C. An injection volume of 5 µL was used. The mass spectrometer was operated in both positive and negative electrospray ionization (ESI) modes to maximize detection sensitivity for a broader range of compounds. Full-scan MS spectra were acquired at a resolving power of 30,000 (at *m*/*z* 200) over the mass range of 100–1200 *m*/*z*, followed by all-ion fragmentation (AIF) for structural elucidation. To maintain instrument and column integrity and to minimize carryover between samples, blank injections of acetonitrile were performed after each sample run.

### 2.5. FPIA of 2,4-D

#### 2.5.1. Choice of Tracer and MAb Working Concentrations

A series of tracer dilutions (0.1–20 nM, 1 mL) were prepared in test tubes in 50 mM borate buffer, pH 8.5 (BB), with the addition of NaN_3_ (0.01%). Intensity of fluorescence and its polarization for the prepared solutions were measured. The concentration dependences were plotted, and working concentrations of the tracer, incorporating factors such as intensity, were selected so that a stable FP signal was maintained.

A tracer working solution (TWS) was prepared in BB, the intensity of which was about 400,000 units/mL. A total of 0.5 mL Mb with different concentrations (0–6 μg/mL) was added to 0.5 mL of TWS. Intensity of fluorescence and its polarization for the prepared solutions were measured after 30 min of incubation at 25 °C. Based on the experimental data, the dependences of MAb concentrations on the bound fraction (Fb) were constructed, and Kd was calculated for the MAb*2,4-D–GAF and MAb*2,4-D–BDF complexes (Supplementary Materials).

#### 2.5.2. Competitive FPIA and Testing Spiked Samples of Juice and Water

For 2,4-D detection in juice and water, a tracer solution (5 nM, 0.5 mL) was added to a series of 2,4-D solutions (0.01–10,000 ng/mL, 0.05 or 0.001–1000 ng/mL, 0.5 mL) and thoroughly vortexed. Then, MAb (5.5 nM (0.83 μg/mL), 0.5 mL or 55 nM (8.3 μg/mL), 0.05 mL) was added, respectively. After 10-15 min incubation of the reaction mixtures, mP was measured.

The juice was bought in supermarkets and stored at 4 °C. Before the FPIA, juice samples were spiked with 2,4-D and analyzed without subsequent sample preparation. Spiked water samples with known concentrations of 2,4-D were obtained, and pH was adjusted to 8.5 and stored at 4 °C. Upon testing samples of juice or water, 0.05 or 0.5 mL of spiked juice or water was mixed with the tracer instead of standard 2,4-D solutions.

### 2.6. Evaluation of the Assay Results and Specificity

The plots of mP (y) versus the 2,4-D concentrations (x) were built and fitted to a four-parameter logistic function. The LODs, cutoffs, and working ranges were evaluated in accordance with the IUPAC recommendations [43].

The specificity of FPIA has been studied under optimal assay conditions. Competitive FPIA was conducted using the following other acids: 2,4,5-chlorophenoxyacetic acid (2,4,5-T), 2,4-chloro-5-fluorophenoxyacetic acid (2,4,5-CFPA), 2-chloro-4-fluorophenoxyacetic acid (2,4-CFPA), pentachlorophenoxyacetic acid (PCPA), and 4-chloro-o-toluoxyacetic acid (4-CTA) in the concentration range 1–1000 ng/mL. Cross-reactivity (CR) was estimated using the following equation:

(1) $$\mathrm{CR}\%=\frac{\mathrm{IC}50_{\mathrm{CR}}}{\mathrm{IC}50_{2,4-D}}*100\%$$

where IC50_CR_ and IC50_2,4-D_ are concentrations of the cross-reactant and 2,4-D, causing 50% inhibition of MAb binding with the tracer.

## 3. Results and Discussion

### 3.1. Fluorescent Tracers

In the study, two derivatives of fluorescein, BDF and GAF, were characterized as fluorophore labels for FPIA. The presence of functional amino groups in the structure of the fluorophore allows its simple conjugation with 2,4-D containing carboxyl group with the formation of an amide bond [36]. BDF is obtained using FITC and an amino group-containing derivative, buthylendiamine, while GAF was a commercial preparation that already had an NH_2_ group.

**Figure 2:**
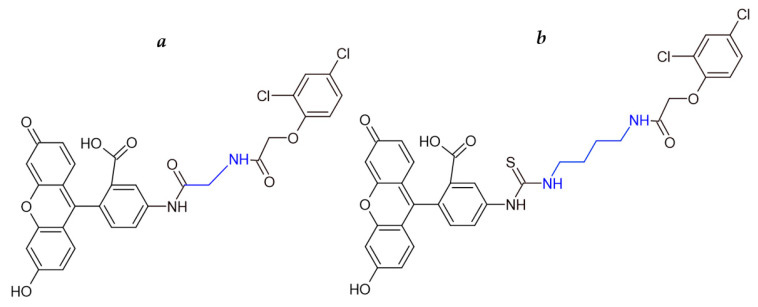
Structure of 2,4-D–GAF (**a**) and 2,4-D–BDF (**b**) tracers. The spacers between fluorescein and 2,4-D are colored blue.

The structures of the 2,4-D–GAF and 2,4-D–BDF tracers are shown in Figure 2. Carbodiimide activation was used to conjugate 2,4-D with BDF and GAF, and the synthetic products were then purified by TLC. Consequently, a fraction with retention factors (Rf) of 0.9 was produced for every tracer.

**Figure 3:**
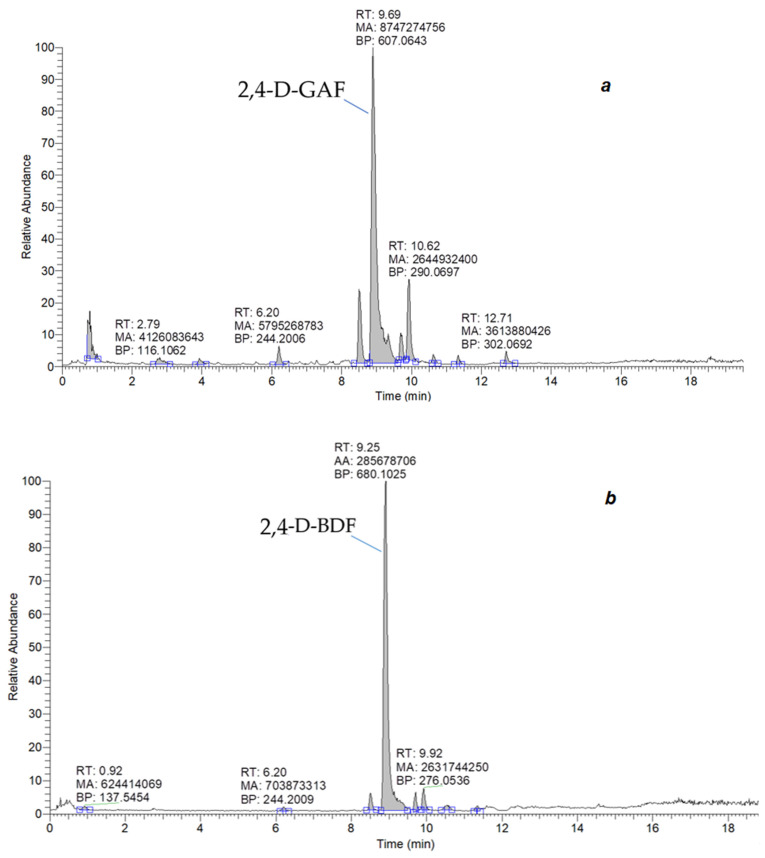
Total ion current (TIC) mass chromatograms of 2,4-D-GAF (**a**) and 2,4-D-BDF (**b**) samples in positive-ion electrospray ionization mode (RT—retention time; MA—manual area; BP—base peak).

The obtained tracers were investigated by mass spectrometry. The results for the new tracers 2,4-D–GAF and 2,4-D–BDF are presented in Figure 3. A mass chromatogram of the first order under the mode of registration of positively charged ions was conducted. The first-order mass spectra in the positive ion detection mode showed peaks corresponding to [M+H]+ (*m*/*z* 607.0643) for 2,4-D–GAF and (*m*/*z* 680.1025) for 2,4-D–BDF, respectively. Thus, the production of fluorescent conjugates 2,4-D–GAF and 2,4-D–BDF was demonstrated. These conjugates had different spacer lengths: one CH_2_ group in 2,4-D–GAF and four CH_2_ groups in 2,4-D–BDF (Figure 2, blue color).

**Figure 4:**
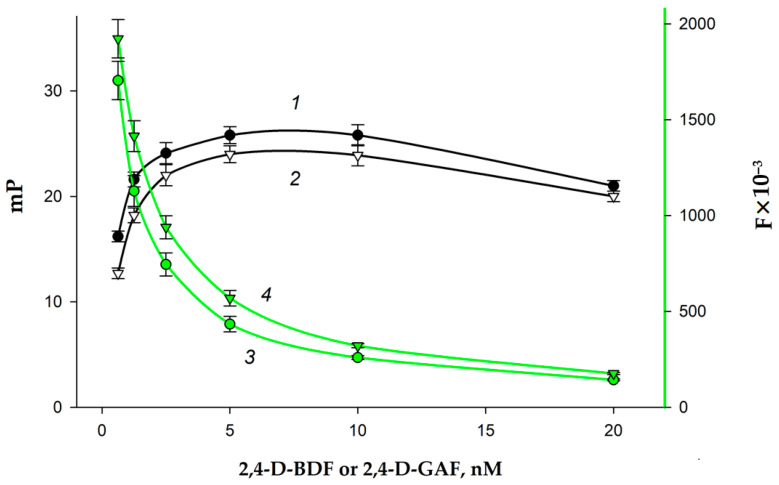
Changes in fluorescence polarization of 2,4-D–BDF (1) and 2,4-D–GAF (2) and fluorescence intensity for 2,4-D-BDF (3) and 2,4-D-GAF (4) of tracers at different concentrations (25 °C; BB; pH 8.5).

We studied the fluorescent properties of the obtained tracers. For this purpose, 2,4-D–BDF and 2,4-D–GAF conjugates were prepared in solutions with different concentrations, and the intensity (F) and mP values were recorded; the results are shown in Figure 4. It is seen that in the fluorescence intensity range from 200,000 to 600,000 U, the FP signal was stable, while the concentrations of all tracers were from 2 to 10 nM. At these concentrations, the tracers gave an optimal signal-to-noise ratio and a stable FP signal.

### 3.2. Antibody Testing

**Figure 5:**
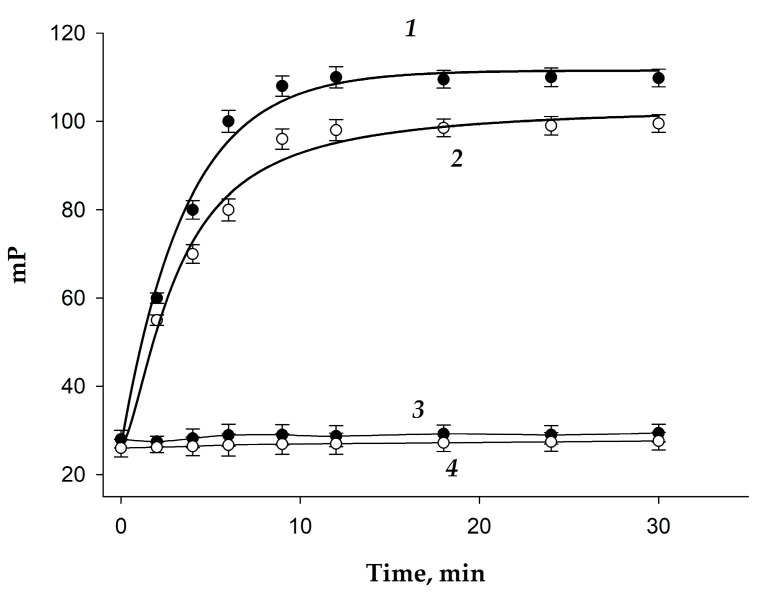
Kinetics of fluorescence polarization change at interaction 2,4-D-GAF (●) and 2,4-D-BDF (o) with specific MAb (4 nM, curves 1 and 2) and non-specific MAb2 (10 nM, curves 3 and 4) (25 °C; BB; pH 8.5).

The binding kinetics of 2,4-D–GAF and 2,4-D–BDF with specific MAb and non-specific MAb2 were studied. To 0.5 mL tracer with concentration 5 nM was added 0.5 mL MAb or MAb2, and changes in mP values were measured within 30 min. The obtained results demonstrated a rapid increase in mP and the achievement of equilibrium in 10 min (mP reaches a plateau) with specific MAb (Figure 5, curves 1 and 2). However, when non-specific Mab2 was added to the 2,4-D–GAF and 2,4-D–BDF tracers, no changes in the fluorescence polarization signal were observed (Figure 5, curves 3 and 4), indicating specific binding of the tracers to the MAb.

### 3.3. Determination Affinity of 2,4-D-BDF and 2,4-D-GAF with MAb

**Figure 6:**
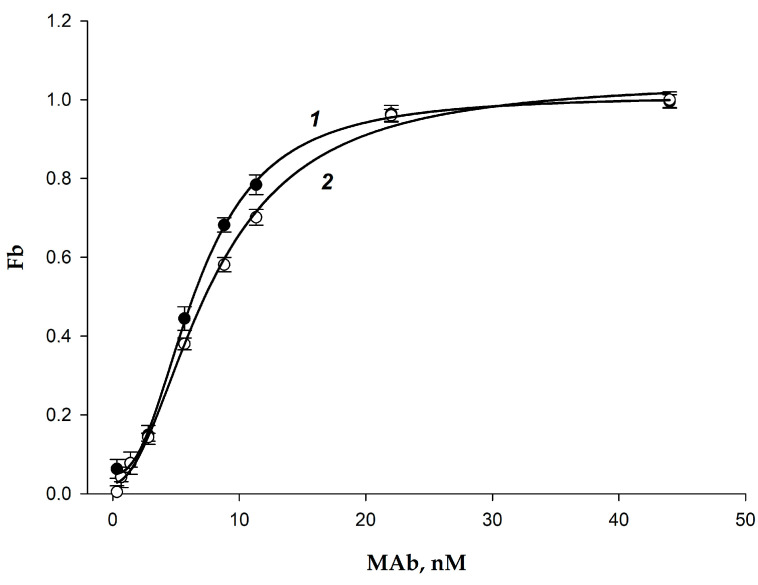
Dependences of the proportion of the bound fraction (F_b_) tracers 2,4-D-BDF (1) and 2,4-D-GAF (2) on the MAb concentration (25 °C; BB; pH 8.5; n = 3).

The FPIA method is known to be useful for characterizing protein/ligand interactions [44,45,46,47]. We studied the affinity of the tracers 2,4-D-BDF and 2,4-D-GAF with MAb by adding 0.5 mL of a 5 nM tracer solution (2,4-D-BDF or 2,4-D-GAF) to 0.5 mL of an antibody solution with different concentrations (0–90 nM), measuring the FP signal after 10–15 min and calculating the proportion of the bound fraction ((F_b_) is the ratio of the bound fraction to the total tracer concentration) (Equation (2)). The results are shown in Figure 6. The interactions were characterized by the following parameters:

(2) $$F_{b}=\frac{C_{x}}{C_{0}}=\frac{mP-mP_{0}}{mP_{max}-mP_{0}}$$

where C_x_ is the concentration of the antibody-bound tracer, C_0_ is the total concentration of the tracer, mP is the measured FP signal, mP_0_ is the polarization of the free tracer, and mP_max_ is the polarization upon complete binding of the tracer to the antibodies.

As can be seen, the dependences of F_b_ on the concentration of MAb for the 2,4-D-BDF and 2,4-D-GAF tracers are similar (Figure 6), which indicates their close affinities to the antibodies. Based on the F_b_ values and Equation (S8) in the Supplementary Materials, dissociation constants of the 2,4-D-BDF*MAb and 2,4-D-GAF*MAb complexes were calculated.

The equilibrium dissociation constants (K_d_) of the tracer complexes with MAb were calculated (see Supplementary Materials) using Sigma Plot 11 [47]. Taking into account the bivalence of antibodies, K_d_ for 2,4-D-BDF and 2,4-D-GAF was 3.2 ± 0.5 and 3.7 ± 0.6 nM, respectively. As can be seen from the presented data, the dissociation constants for the two tracers with antibodies are close, which is an advantage of the chosen modification strategy since chemical modification is often accompanied by a change in the binding constants [39,48]. In addition, it is evident that the obtained dissociation constants do not depend on the length of the spacer, which indicates that the modification does not affect the binding of the tracer to antibodies.

### 3.4. Development of 2,4-D FPIA 

**Figure 7:**
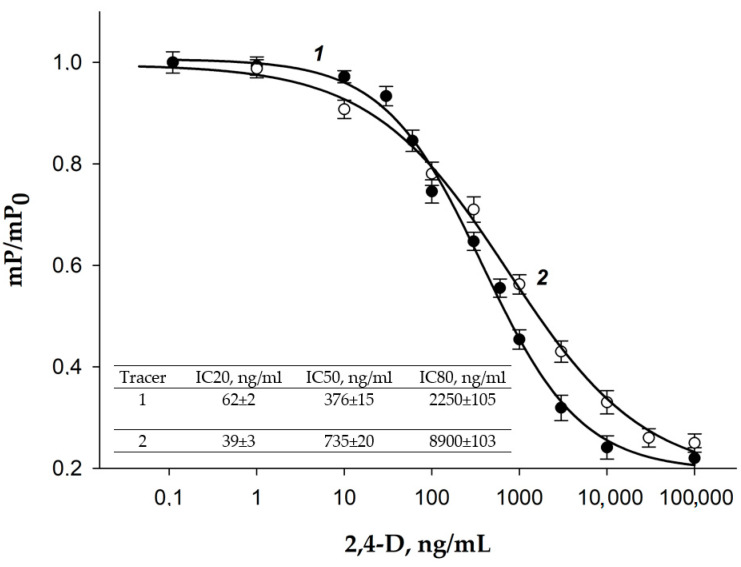
Dependencies of the ratios of changes in fluorescence polarization signals (mP/mP_0_) on the concentration of 2,4-D for 2,4-D-BDF (1) and 2,4-D-GAF (2) tracers. The final concentration of the tracers was 2.5 nM, and the MAb concentration was 0.5 µg/mL (3,3 nM), n = 2.

Two tracers, 2,4-D-GAF and 2,4-D-BDF, with different spacer lengths between the analyte and fluorescein, were produced. 2,4-D-GAF had a shorter spacer (one CH_2_ group), while 2,4-D-BDF had four CH_2_ groups. To select the tracer for sensitive competitive FPIA, we obtained dependances of changing FP signal from 2,4-D concentration and compared them normalized dependences of mP/mP_0_ for 2,4-D-BDF и 2,4-D-GAF (Figure 7) at the same MAb concentration (0.5 µg/mL (3.3 nM)). Analytical characteristics of the curves for 2,4-D determination for tracers 2,4-D-BDF и 2,4-D-GAF are presented in Figure 7. As shown, more sensitive FPIA was obtained using the 2,4-D-GAF tracer, which had a shorter spacer. The use of a tracer with a short spacer would make the analysis more sensitive due to steric hindrances and electrostatic repulsion of the fluorescent label by the charged groups of the antigen-binding center of the antibody. It has been shown that as the length of the spacer decreases, the degree of displacement of the labeled antigen by the unlabeled one increases [49]. Thus, using the 2,4-D-GAF tracer, we obtained a more sensitive assay and a wider detection range (IC20-IC80). Therefore, we further optimized the conditions for the FPIA method for the 2,4-D-GAF/MAb immunoreagent pair.

The selected concentration ensures a high analytical signal for accurate and reproducible analysis on the one hand and allows for the achievement of sensitive 2,4-D detection on the other hand. Considering these factors, the MAb concentration in the reaction solutions should be about K_d_ (Figure 6). The optimal concentration of MAb for sensitive FPIA was fixed as 2.7 nM (finish concentration in the reaction mixture). The incubation time of 10–15 min was chosen since, when varying the concentrations of standard 2,4-D solutions, the equilibrium was established in all reaction mixtures (Figure 5, curve 1).

**Figure 8:**
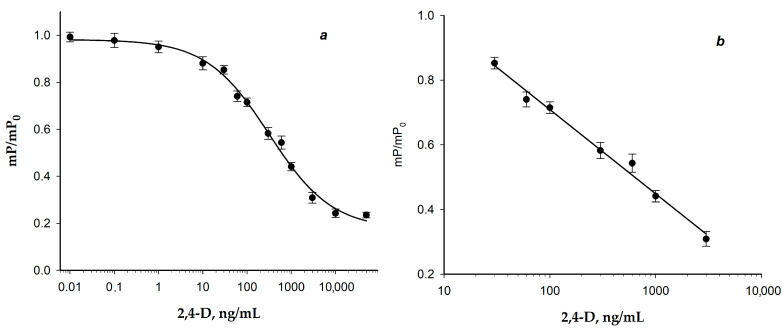
Calibration curve of 2,4-D in FPIA (**a**) and its linear range (**b**). The final concentrations of the reactants are [2,4-D-GAF] = 2.5 nM, [MAb] = 0.4 µg/mL (2.7 нM) (25 °C; BB; pH = 8.5). Linear fitting for the figure (**b**) is based on the following equation: Y = (1.23 ± 0.02) *−* (0.26 ± 0.01) × X (R^2^ = 0.98; n = 3).

Based on the competition between the tracer and the detected antigen for the few antibody binding sites, a competitive FPIA was carried out under the chosen circumstances. More labeled antigens stay in the reaction mixture, and, as a result, the measured mP value decreases with increasing concentration of the detected analyte in the sample. To achieve final concentrations of 2.5 and 2.7 nM, respectively, 0.5 mL tracer (2,4-D-GAF) and 50 µL of standard solution were combined with 0.5 mL MAb. The obtained calibration curve of the 2,4-D assay and its linear range are presented in Figure 8.

The developed FPIA enables 2,4-D determination with the LOD of 8 ng/mL (LOD was calculated as IC_10_) and the working range of detectable concentrations of 30–3000 ng/mL. The duration of the measurements was 15 min.

### 3.5. Study of the FPIA Specificity

The specificity of the developed FPIA was assessed by testing the cross-reactivity (CR) with the relevant toxins, which may contaminate foods and environmental waters. The CR (%) was calculated as indicated in Section 2.6 (Equation (1)). The substances similar in structure to 2,4-D were selected as cross-reactants: 2,4,5-trichlorophenoxyacetic acid (2,4,5-T), 2,4-chloro-5-fluorophenoxyacetic acid (2,4,5-CFPA), 2-chloro-4-fluorophenoxyacetic acid (2,4-CFPA), pentachlorophenoxyacetic acid (PCPA), and 4-chloro-o-toluoxyacetic acid (4-CTA). In ELISA, this MAb showed no cross-reactivity with other plant growth regulators with similar structures to 2,4-D [41]. The found CR% for these analogs of 2,4-D (2,4,5-T, 2,4,5-CFPA, and 4-CTA) were less than 20% for FPIA (Table S1). For 2,4-CFPA, the CR% was about 7%, and for PCPA was less than 0.001%. These results indicate that the developed FPIA is characterized by high specificity towards 2,4-D.

### 3.6. Determination of 2,4-D in Juice

The developed FPIA was approbated for the determination of 2,4-D in apple juice. Preliminary testing of all studied samples using the commercial 2,4-D ELISA kits (XEMA) revealed no 2,4-D content. The juice has complex matrices: sugar or sweeteners, food colorings, preservatives, flavorings, antioxidants, and other food additives. The given compounds could affect the results of the homogeneous FPIA that is implemented in one stage without any separations. The juice samples were spiked with 2,4-D at the concentrations selected from the working range and analyzed by the FPIA. The result of the recovery values presented in Table 1 are in the range of 95–110%. These results show that using the developed FPIA of 2,4-D can be revealed for the juice.

### 3.7. Determination of 2,4-D in Water

Water pollution with herbicides is a serious problem worldwide. Homogeneous FPIA is able to quickly and selectively determine the level of toxicants in water. Usually, FPIA does not require special sample preparation when determining toxicants. It is possible to vary the volumes of added reagents to achieve lower detection limits. When analyzing drinking water or water from open reservoirs, there is no need for strong sample dilution; it is enough to simply filter the sample from large particles and bring the pH to that used in the analysis. 

**Figure 9:**
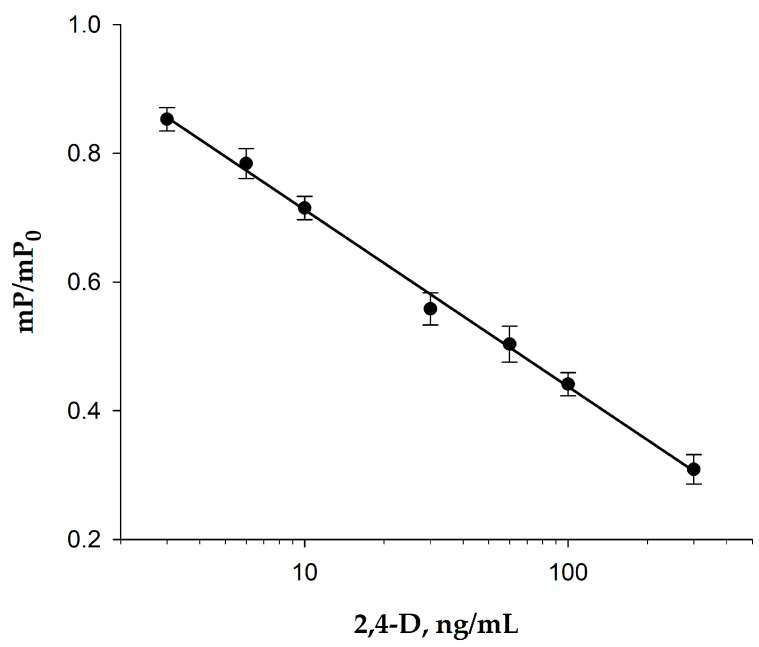
Detection range of the calibration curve of 2,4-D by FPIA in water samples. The final concentrations of the reactants are [2,4-D-GAF] = 2.5 nM, [MAb] = 0.4 µg/mL (2.7 нM) (25 °C; BB; pH 8.5; n = 3) and Y = (0.98 ± 0.02) *−* (0.27 ± 0.01) × X (R^2^ = 0.99; n = 3).

For water analysis, we attempted to reduce the sample dilution up to two times and constructed a new calibration dependence in which we changed the volumes of analyzed samples and immunoreagents. A total of 0.5 mL tracer with a concentration of 5 nM and 0.5 mL of water sample were added to 50 μL of MAb with a concentration of 55 nM. The resulting calibration dependence is shown in Figure 9. The detection limit was determined by the IC_10_ method and was 0.4 ng/mL. The detection (linear) range was 3.3-304 ng/mL, which was determined according to recommendations [43]. This detection range is based on the accurate measurement of the concentration with errors less than 10%.

This calibration dependence (Figure 8) was used to check the FPIA by the recovery test of water samples. This detection limit is almost 100 times lower than the maximum permissible concentration (30 ng/mL), which will allow the detection of the pollutant in water with high sensitivity.

We conducted a recovery test to determine 2,4-D in water. Contaminated samples were made with known concentrations of 5, 30, and 100 ng/mL. The results of the recovery test are presented in Table 1. The recovery of 2,4-D in water samples is in the range of 95–120%.

### 3.8. Comparison of the Obtained Results with Other Investigations

As it was noted above, FPIA and ELISA dominate among the described immunoassays of 2,4-D. Table 2 summarizes data on the sensitivity, assay duration, and types of matrices tested by the new and earlier-developed 2,4-D immunoassays.

The developed FPIA method for determining 2,4-D in juice has a higher detection limit than known ELISA methods, even using the same antibodies [41]. However, it is important to note that the dilution of the sample for determining 2,4-D in juice is 20 times higher, and this dilution is sufficient to eliminate the matrix effect and conduct FPIA without preliminary sample preparation. When determining low concentrations of the herbicide in water, as, for example, may occur in natural reservoirs, we can reduce the sample dilution up to two times with minimal sample preparation (filtration and adjusting the pH to the assay buffer), which sharply reduces the detection limit to 0.4 ng/mL, and it becomes comparable with the LOD obtained in ELISA with the same MAb [41].

Despite the fact that ELISA methods are more sensitive [31,41,53,54] than FPIA, they are inferior in terms of analysis duration, labor intensity, and the need to obtain a calibration dependence for each experiment. From the presented data, it is evident that with the monoclonal antibodies used in this work, it was possible to reduce the detection limit of 2,4-D by one order of magnitude in water compared to other results [37]. Thus, it is shown that the developed FPIA can serve as an effective alternative to classical ELISA for rapid, simple, sensitive, and reproducible analysis of 2,4-D in food products and water.

## 4. Conclusions

Highly sensitive FPIA was designed for the detection of the herbicide 2,4-D. FPIA can be performed within 20 min in one stage by simple incubation of the analyte, specific antibodies, and the tracer. The FPIA method is homogeneous and usually does not require special sample preparation; therefore, when determining the antigen in different samples, it is possible to vary the volumes of added reagents to achieve lower detection limits. For example, juice is a fairly complex multicomponent matrix. The components included in its composition can affect the analysis, and it is most convenient to use small volumes of the added sample, and, as a rule, dilution by 20 times removes the matrix effect completely. For the determination of 2,4-D in juice, we optimized the FPIA using small sample volumes. In order to minimize sample preparation of the analyzed juice samples, we used small volumes (50 μL). The 20-fold dilution of juice in the reaction mixture completely eliminated the matrix effect of the components present in the juice. The achieved 2,4-D LOD was 8 ng/mL for juice. The developed FPIA was successfully applied to determine 2,4-D in apple juice, with a recovery of 95–110%.

When analyzing drinking water or water from open reservoirs, there is no need for strong dilution of the sample; it is enough to simply filter the sample and bring the pH to that used in the analysis; thus, when analyzing water, we can vary the volume of the sample to increase the sensitivity of the analysis. For the determination of 2,4-D in water samples, we optimized the FPIA conditions by increasing the volume of the analyzed sample. Preparing a water sample requires only the separation of large particles and adjustment of the pH of the solutions to the pH of the analyzed buffer. The achieved 2,4-D LOD was 0.4 ng/mL for water. The developed FPIA was successfully applied for the determination of 2,4-D in water with a recovery of 95–120%. This approach can be recommended as an analytical tool for the fast and reliable detection of herbicides in foods. However, the analysis of herbicides in food products with a more complex matrix, such as eggs, dairy, or meat products, may require more serious sample preparation. The use of a portable analyzer enables out-of-laboratory control of 2,4-D content.

## Figures and Tables

**Table 1:** Recoveries of 2,4-D from apple juice and water for the developed FPIA. n = 3.

	№	Added 2,4-D, ng/mL	Found 2,4-D, ng/mL	Recovery, %
apple juice	1	300	320 ± 2	106.6
	2	200	190 ± 2	95.0
	3	30	32 ± 1	106,6
	4	10	11 ± 1	110.0
water	1	100	95 ± 2	95
	2	30	33 ± 1	110
	3	5	6 ± 1	120

**Table 2:** Comparison of the developed and the existing bioanalytical techniques for 2,4-D detection.

Assay Format	Analyte-Binding Reactant	Limit of Detection	Time of the Assay	Samples Tested	Reference
FPIA	PAb *	2 μg/g	5–7 min	Soil	[50]
FPIA	MAb	100 ng/mL	7 min	Grain	[35]
FPIA	MAb	40 ng/g	1 min	Cereals	[36]
LFIA	MAb	20 ng/mL	15 min	Vegetables	[41]
Quartz crystal microbalance	MAb	13 ng/mL	120 min		[51]
LFIA	Nanobodies	11 ng/mL	15 min	Water, fruits, vegetables	[52]
FPIA	MAb	4 ng/mL	8-12 s	Water, juice, wine	[37]
Chemiluminescent ELISA	PAb	3 ng/mL	30 min	_	[53]
Chemiluminescent ELISA	MAb	2 ng/mL	60 min	Fruits	[31]
Fluorescent ELISA	PAb	1.2 ng/mL	ND	Water	[54]
LFIA with Fe-single-atomic site catalyst	MAb	0.82 ng/mL	60 s	Urine	[55]
ELISA	MAb	0.267 ng/mL	120 min	Vegetables	[41]
FPIA	MAb	0.4 ng/mL 8 ng/mL	20 min	Water, juice	This work

* PAb—polyclonal antibodies.

## Data Availability

Data are available upon request from authors.

## Supplementary Materials

The following supporting information can be downloaded at: https://www.mdpi.com/article/10.3390/bios15010032/s1, Derivation of the equation for calculating K_d_; Table S1: Data on MAb cross-reactivity (CR) for FPIA.
