# Supplementary material for: Fluorescence Polarization Immunoassay for Rapid, Sensitive Detection of the Herbicide 2,4-Dichlorophenoxyacetic Acid in Juice and Water Samples
Source: Biosensors (Basel). 2025 Jan 9;15(1):32. doi: 10.3390/bios15010032 (PMC11763590; doi:10.3390/bios15010032)
Supplement: Supplementary file 1 [file biosensors-15-00032-s001.zip › biosensors-3365425-supplementary.pdf]

biosensors

MDPI

Supplement

Fluorescence Polarization Immunoassay for Rapid, Sensitive Detection of the Herbicide 2,4-Dichlorophenoxyacetic Acid in Juice and Water Samples

Liliya I. Mukhametova [1,2,*], Marya K. Kolokolova [1], Ivan A. Shevchenko [1], Boris S. Tupertsev [3], Anatoly V. Zherdev [2], Chuanlai Xu [4] and Sergei A. Eremin [1,2,*]

[1] Faculty of Chemistry, M.V. Lomonosov Moscow State University, Leninsky Gory 1/3, 119991 Moscow, Russia; kolokolovamasha@yandex.ru (M.K.K.); ivansevcekno@gmail.com (I.A.S.)
[2] A. N. Bach Institute of Biochemistry, Research Center of Biotechnology of the Russian Academy of Sciences; Leninsky Prospect 33, 119071 Moscow, Russia; zherdev@inbi.ras.ru
[3] N.N. Semenov Federal Research Center for Chemical Physics, Russian Academy of Sciences, Kosygina 4, 119991 Moscow, Russia; btoupersev@gmail.com
[4] School of Food Science and Technology, Jiangnan University, Wuxi 214122, China; xcl@jiangnan.edu.cn
* Correspondence: liliya106@mail.ru (L.I.M.); eremin_sergei@hotmail.com (S.A.E.)

The FPIA method is suitable for characterizing antibody/ligand interactions. Equation (S1) describes the reaction of conjugate binding 2,4-D–BDF or 2,4-D–GAF (2,4-D–FLU) with MAb:

$$\text{MAb} + \text{2,4-D–FLU} \rightleftharpoons (\text{MAb*2,4-D–FLU}) \qquad \text{(S1)}$$

Received: 29 November 2024
Revised: 31 December 2024
Accepted: 7 January 2025
Published: 9 January 2025

Citation: Mukhametova, L.I.; Kolokolova, M.K.; Shevchenko, I.A.; Tupertsev, B.S.; Zherdev, A.V.; Xu, C.; Eremin, S.A. Fluorescence Polarization Immunoassay for Rapid, Sensitive Detection of the Herbicide 2,4-Dichlorophenoxyacetic Acid in Juice and Water Samples. *Biosensors* **2025**, *15*, 32. https://doi.org/10.3390/bios15010032

Copyright: © 2025 by the authors. Licensee MDPI, Basel, Switzerland. This article is an open access article distributed under the terms and conditions of the Creative Commons Attribution (CC BY) license (https://creativecommons.org/licenses/by/4.0/).

The dissociation constant Kd for the MAb*2,4-D–FLU complex is expressed as follows:

$$K_d = \frac{[MAb]*[2{,}4-\mathrm{D}-\mathrm{FLU}\,]}{[\mathrm{MAb}*2{,}4-\mathrm{D}-\mathrm{FLU}\,]} \qquad \text{(S2)}$$

Provided that the initial concentrations of MAb and fluorescently labeled 2,4-D–FLU conjugate are close, the dynamic concentrations of MAb and 2,4-D–FLU accord to the following equations:

$$[\mathrm{MAb}] = [\mathrm{MAb}]_0 - [\mathrm{MAb} * 2{,}4 - \mathrm{D}-\mathrm{FLU}\,] \qquad \text{(S3)}$$
$$[2{,}4 - D - BDF] = [2{,}4 - \mathrm{D}-\mathrm{FLU}\,]_0 - [\mathrm{MAb} * 2{,}4 - \mathrm{D}-\mathrm{FLU}\,] \qquad \text{(S4)}$$

In this case, equation (S2) takes the form:

$$K_d = \frac{([\mathrm{MAb}]_0 - [\mathrm{MAb}*2{,}4-\mathrm{D}-\mathrm{FLU}\,])*([2{,}4-\mathrm{D}-\mathrm{FLU}\,]_0 - [\mathrm{MAb}*2{,}4-\mathrm{D}-\mathrm{FLU}\,])}{[\mathrm{MAb}*2{,}4-\mathrm{D}-\mathrm{FLU}\,]} \qquad \text{(S5)}$$

If we denote the variable concentration of the [MAb*2,4-D–FLU] complex by the symbol Cx, then

$$K_d = \frac{([MAb]_0 - C_x)*([2,4\text{-}D\text{-}FLU\ ]_0 - C_x)}{C_x} \quad (S6)$$

The proportion of MAb bound to the 2,4-D–FLU tracer, the Fb value (the ratio of the concentration of the antibody-bound tracer Cx to the initial concentration of the tracer 2,4-D–FLU - C_0) is determined as:

$$F_b = \frac{C_x}{C_0} = \frac{mP - mP_0}{(mP_{max} - mP)*Q + (mP - mP_0)} \quad (S7)$$

where mP is the observed degree of sample polarization, mP_0 is the degree of polarization of the free fluorescently labeled 2,4-D–BDF or 2,4-D–GAF conjugate, mPmax is the degree of polarization of the MAb*2,4-D–BDF or MAb*2,4-D–GAF complexes upon complete binding, and Q is the ratio of the fluorescence intensities of the bound to the free tracer.

When measuring the fluorescence polarization signal of studied solutions, the fluorescence intensity of the MAb*2,4-D–BDF or MAb*2,4-D–GAF complexes does not change upon binding of tracer to antibody, therefore Q = 1. Expression (S6) is transformed into a quadratic equation, and Cx is calculated as one of its roots. The transformed equation (S6) can be substituted into expression (S7) we obtain:

$$F_b = \frac{mP - mP_0}{mP_{max} - mP_0} = \frac{a - \sqrt{a^2 - 4*C_0*[MAb]_0}}{2*C_0} \quad (S8)$$

where $a = K_d + [2,4 - D\text{-}\ FLU\ ]_0 + [MAb]_0$.

$$mP - mP_0 = (mP_{max} - mP_0)\frac{a - \sqrt{a^2 - 4*C_0*[MAb]_0}}{2*C_0} \quad (S9)$$

Taking the bivalence of antibodies into account, we considered the protein concentration doubled and calculated Kd by equation (S9) using the Sigma Plot 11 program (Systat Software Inc., Palo Alto, CA, USA).

Table S1. Data on MAb cross-reactivity (CR) for FPIA.

**Substance**	**Structure**	**CR, %**
2,4-D	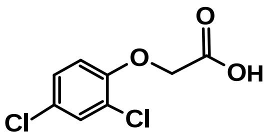	100
2,4,5-T	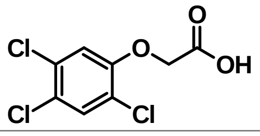	15.6
2,4,5-CFPA	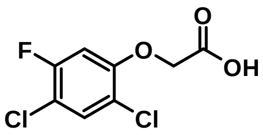	16.4

4-CTA	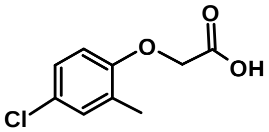	18.8
2,4-CFPA	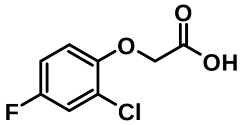	6.8
PCPA	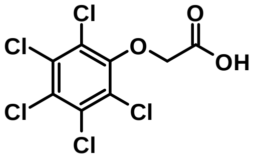	<0.001

Disclaimer/Publisher's Note: The statements, opinions and data contained in all publications are solely those of the individual author(s) and contributor(s) and not of MDPI and/or the editor(s). MDPI and/or the editor(s) disclaim responsibility for any injury to people or property resulting from any ideas, methods, instructions or products referred to in the content.
